# Prospective intra/inter-observer evaluation of pre-brachytherapy cervical cancer tumor width measured in TRUS and MR imaging

**DOI:** 10.1186/s13014-019-1352-7

**Published:** 2019-10-04

**Authors:** Mario Federico, Carmen Rosa Hernandez-Socorro, Ivone Ribeiro, Jesus Gonzalez Martin, Maria Dolores Rey-Baltar Oramas, Marta Lloret Saez-Bravo, Pedro Carlos Lara Jimenez

**Affiliations:** 1 Radiation Oncology Department, Hospital Universitario de Gran Canaria Dr. Negrín, Barranco de la Ballena s/n, 35010 Las Palmas de Gran Canaria, Spain; 2 Radiology Department, Hospital Universitario de Gran Canaria Dr. Negrín, Las Palmas de Gran Canaria, Spain; 3 Unidad de Investigación, Hospital Universitario de Gran Canaria Dr. Negrín, Las Palmas de Gran Canaria, Spain

**Keywords:** Cervical Cancer, Image guided brachytherapy, Ultrasound, TRUS, Magnetic resonance, 3D brachytherapy

## Abstract

**Background:**

Ultrasound (US) imaging has been proved as an excellent diagnostic tool in gynecology and, due to its wide availability and limited cost, is under intense investigation as base for dose adaptation in cervical cancer brachytherapy. Purpose of this work is to test inter/intra-observer uncertainties between magnetic resonance (MR) and trans-rectal ultrasound (TRUS) imaging in defining maximum tumor width before first brachytherapy (BT) application in a prospective cohort of cervical cancer patients undergoing image-guided adaptive brachytherapy (IGABT).

**Methods:**

One hundred ten consecutive cervical cancer patients treated between 2013 and 2016 were included. Before the first BT implant patients underwent MR and TRUS scan with no applicator in place. Images were independently analyzed by three examiners, blinded to the other’s results. With clinical information at hand, maximum tumor width was measured on preBT TRUS and MR. Quantitative agreement analysis was undertaken. Intra-class correlation coefficient (ICC), Passing-Bablok and Bland Altman plots were used to evaluate the intra/inter-observers measurement agreement.

**Results:**

Average difference between tumor width measured on MR (HRCTV_MR_) and TRUS (HRCTV_TRUS_) was 1.3 ± 3.2 mm (*p* <  0.001); 1.1 ± 4.6 mm (*p* = 0.01) and 0.7 ± 3 mm (*p* = 0.01). The error was less than 3 mm in 79, 82 and 80% of the measurements for the three observers, respectively. Intra-observer ICC was 0.96 (CI95% 0.94–0.97), 0.93 (CI95% 0.9–0.95) and 0.96 (CI95% 0.95–0.98) respectively. Inter-observer ICC for HRCTV_MR_ width measures was 0.92 (CI95% 0.89–0.94) with no difference among FIGO stages. Inter-observer ICC for HRCTV_TRUS_ was 0.86 (CI95% 0.81–0.9). For FIGO stage I and II tumors, ICC HRCTV_TRUS_ values were comparable to respective HRCTV_MR_ ICC values. For larger tumors HRCTV_TRUS_ inter-observer ICC values were lower than respective HRCTV_MR_ although remaining acceptable.

**Conclusions:**

Our results suggest that TRUS is equivalent to MR in assessing preBT tumor maximum width in cervical cancer FIGO stage I/II. In more advanced stages TRUS seems to be slightly inferior to MR although maintaining a good agreement to gold standard imaging.

**Electronic supplementary material:**

The online version of this article (10.1186/s13014-019-1352-7) contains supplementary material, which is available to authorized users.

## Background

Cervical cancer is the fourth most common cancer in women worldwide and the eight overall. A large majority (around 85%) of the global burden occurs in the less developed regions [1]. For decades radiotherapy (RT) has been the standard of care for locally advanced disease and brachytherapy (BT) is an essential part of the treatment [2, 3]. In the last decade, 3D treatment planning was introduced for cervical cancer BT [4–9] with outstanding clinical results [10–14]. The goal of 3D adaptive BT is to tightly shape the radiation dose to the individual patient’s anatomy and tumor topography for each BT fraction, with the intent to deliver 85–90 Gy_EQD2_ (radiobiologically equieffective dose of 2 Gy per fraction) to the tumor while minimizing the dose to organs at risk (OAR). The precondition for safe BT treatment individualization is the precise identification of target volumes. Magnetic Resonance (MR) has clear advantages in terms of image quality [15] as it allows the optimal definition of normal peri-cervical soft tissues, tumor extension within the cervix, parametrial infiltration and topography. Additionally, MR enables 4D volume adaptation following tumor regression during external beam radiotherapy (EBRT) [16, 17]. Unfortunately, due to its costs and limited availability, the majority of patients worldwide are precluded from receiving MR-based BT treatment [18, 19]. Computer tomography (CT) imaging alone is not an alternative to MR because it’s poor soft tissue contrast is inadequate to precisely define cervical tumors [20] and burdens of parametrial infiltration [21, 22]. Approaches with less intense MR routine like hybrid MR/CT protocols have been investigated with promising results [23] but still rely on some MR imaging. TRUS has excellent soft tissue resolution, is affordable, and has been used extensively in cervical cancer diagnosis [24]. Moreover, TRUS has been used to aid in proper BT applicator insertion and guidance, and for correct placement of parametrial needles, since it is, among all ultrasound (US) modality, the one that better depicts parametrial infiltration. For all these reasons TRUS is under investigation as a potential alternative to MR for Image-Guided Adaptive Brachytherapy (IGABT) planning [25, 26]. Some shortcomings may however limit TRUS use, such as the intrinsic operator dependency, the inadequate visualization of tumor regression areas in the parametria and lastly, the difficulty in evaluating the relationship of extensive tumors to the pelvic sidewall when the edge of infiltration is beyond the range of the probe. The aim of the present study is a blinded multi-observers comparison of TRUS and MR, assessing tumor maximum width before first BT application (without applicator in place) in a large cohort of cervical cancer patients undergoing IGABT.

## Methods

### Patient characteristics and diagnostic workup

After ethical committee approval, between 2013 and 2016, 110 consecutive biopsy-proven cervical cancer patients referred to our department were prospectively included. Clinic and pathological features are given in Table 1. Median age was 52.7 year (23.8–88.6) with a vast majority of patients having locally advanced tumors. 54.5% had tumor width at diagnosis (measured on MR images) larger than 5 cm.

**Table 1:** Patient characteristics

Characteristic	
Age (years)	
Median	52,7
Range	23.8–88.6
Histology (N, %)	
Squamous cell carcinoma	84 (76.3%)
Adenocarcinoma	19 (17.3%)
Adenosquamous cell carcinoma	5 (4.6%)
Clear cell carcinoma	1 (0.9%)
Carcinosarcoma	1 (0.9%)
FIGO stage (N, %)	
IB	19 (17.3%)
IIB	67 (60.9%)
IIIB	14 (12.7%)
IVA	10 (9.1%)
MR tumor width at diagnosis (N, %)	
< 5 cm	50 (45.5%)
> 5 cm	60 (54.5%)

*Abbreviations*: *N* Number of patients, *FIGO* International Federation of Gynaecology and Obstetrics, *MR* Magnetic Resonance

The diagnostic workout consisted of a thorough clinical examination and TRUS imaging acquisition. Clinical findings were reported on a clinical drawing chart. Furthermore, patients underwent thoracic-abdominal contrasted CT and pelvic MR scan. Patients younger than 70 and without obvious macroscopic para-aortic node involvement on CT/MR imaging underwent laparoscopic retroperitoneal para-aortic lymphadenectomy [27]. Patients excluded from surgical nodal staging underwent ^18^FDG PET-CT scan.

### Treatment

After CT simulation (Somaton Sensation Open multislice scanner, Siemens), patients received pelvic 3D conformal EBRT (1.8 Gy per fraction up to 45 Gy total dose), with concomitant chemotherapy (weekly intravenous cisplatin, 40 mg/m^2^) when feasible. Para-aortic nodes were included in the RT field in case of histologically proven involvement or positive PET-CT scan.

BT schedule consisted of 4 Ir192 high dose-rate (HDR) intracavitary or intracavitary/interstitial BT fractions of 7 Gy each, within 2 different implants with an interval 7–10 days between insertions. BT insertions were performed under spinal or general anesthesia. BT applicators were MR compatible tandem-ovoids (Utrecht Interstitial CT/MR Applicator, Elekta) or in-house modified MR compatible vaginal cylinder with a perineal template for cases with extended tumor involvement of lower vagina. The interstitial component consisted of plastic needles (ProGuide round tip needles, Elekta) or round tip titanium needles (Elekta). After the first BT insertion patients underwent 1.5 T MR scan with a phase-array surface pelvic coil (T2 FSE sequences in paraxial, coronal and sagittal plane orientation with a slice thickness of 3.5 mm) for applicator reconstruction and dosimetry. The MR scanner was a Magnetom Espree 1.5 T, Siemens. Furthermore, patients underwent a CT scan (2 mm slice thickness) for research purposes. Images were transferred to Oncentra Brachytherapy planning system (Oncentra Brachytherapy v.4.1, Elekta). Target volumes and OARs were contoured according to GEC-ESTRO recommendations [4, 5] and dose optimized to the HRCTV and OARs.

### Pre-BT tumor assessment

One or two days before the first BT insertion, all patients underwent MR scan (preBT MR), which was used for BT application pre-planning. T2 FSE sequences with a phase-array surface pelvic coil (5 mm slices thickness) in axial and paraxial (adjusting the angle of acquisition to the uterus position in order to obtain a paraxial plane perpendicular to uterine axis), sagittal and coronal orientation were taken with 1 T (Panorama 1 T open MR system, Philips Medical System) or 1.5 T (Siemens Magnetom Espree 1,5 T MRI System) units, depending on the availability. Images were stored on Oncentra Brachytherapy workstation. Furthermore, patients underwent physical examination and clinical features were reported on clinical drawings.

### Patients’ preparation

To maximize the quality of TRUS imaging a thorough patient preparation protocol was followed. In details: starting 3 days before the scheduled brachytherapy procedure, all patients were instructed to follow a low fiber and low carb diet; the night before brachytherapy procedure, patients were admitted to the hospital and underwent a bowel preparation protocol consisting of liquid diet and 2 subsequent enemas.

### Trans-rectal ultrasound acquisition

The radiation oncologist in the operating theatre, right before the BT procedure, obtained TRUS images (preBT TRUS) taken with patients under anesthesia, without the applicator in place and according to a standardized protocol. In details: before TRUS image acquisition, a 5/7 F Foley catheter (Histerosonography - Histerosalpingography catheter, Cooper Surgical, USA) was introduced into the uterus in order to visualize cervical canal and the Foley balloon filled with 3–4 cc of sterile saline solution and pulled back up to the uterine internal orifice; bladder was filled with 100 cc of saline solution; bowel preparation was routinely verified as part of the brachytherapy procedure and, in case of poor preparation, a lower bowel irrigation was performed.

The transrectal probe (Hitachi EUP-U533 biplane radial/linear probe 5–10 MHz) was covered with a lubricated protective sheath, fixed to a ultrasound stepper unit (OncoSelect Stepper, Elekta) and inserted into the rectum. Before image acquisition, the position of transrectal probe was adjusted in order to be parallel to the uterus. This was achieved tilting and rotating freely the probe angle in all directions to overcome recto-sigmoid junction and adjust to the individual patient uterine position in order to maintain the TRUS probe parallel to the uterus. The intrauterine histerosalpingosonography catheter improved the visualization of cervical canal and uterine axis, and helped to define the uterine internal orifice and the transition between upper cervix and lower uterine corpus. Transrectal probe was inserted as much as possible in order to fully visualize the *fundus uteri*. From this position, a 3D image acquisition was performed with a manual pull-back (1 mm step) of the TRUS probe in the stepper unit from the *fundus uteri* to the lower third of the vagina. The US scanner was a Hitachi EUB 5500. Images were stored and analyzed on Oncentra Prostate workstation (Oncentra Prostate v4.2, Elekta).

### Study design, image analysis and measurement procedure

PreBT MR and preBT TRUS imaging were independently analyzed by three examiners: two radiation oncologists (MF and IR) fully dedicated to brachytherapy, and a US-dedicated radiologist (CRHS). At the time of preBT TRUS imaging analysis, observers were blind to MR images. Moreover, each observer was blind to the other observer’s results. First, clinical drawings at diagnosis and at the time of BT were evaluated. With clinical information at hand, preBT TRUS images were analyzed and tumor maximum width measured (HRCTV_TRUS_). Finally, the maximum tumor width was measured on preBT MR (HRCTV_MR_).

HRCTV_TRUS_ was defined on gray scale levels as the solid cervical mass, hypoechoic in respect to normal parametrium, with eventual continuous extension into parametrial space [24].

**Fig. 1:**
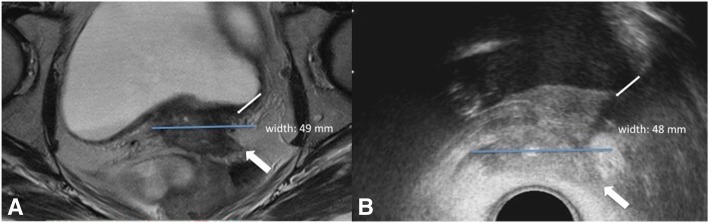
A case of cervical cancer FIGO stage IVA because bladder infiltration. In preBT MR (**a**) tumor width is 49 mm. In preBT TRUS (**b**) tumor width is 48 mm. Thin arrows show posterior bladder wall invasion. Thick arrows show parametrial invasion

HRCTV_MR_ was defined as the macroscopic residual tumor extension visualized on T2 weighted MR, as high signal intensity mass, plus potential surrounding parametrial “grey zones” with intermediate signal intensity in the area of the initial tumor infiltration and the remaining low signal intensity cervical stroma [10]. An example is provided in Fig. 1.

The maximum width measurement was performed as follow: the 3D preBT TRUS imaging was revised and the cranio-caudal axis of the uterus was identified using the cervical canal depicted by the uterosalpingography catheter as reference. The maximum HRCTV_TRUS_ width was measured perpendicular to the uterine axis, along the horizontal transverse axis. The same procedure was followed for the measurement of maximum HRCTV_MR_ width.

### Statistical analysis

Descriptive statistics, data analysis, and plots were performed with R Core Team (2014) and Prism 6.0 (2015) software. A Wilcoxon paired *t-test* was used to compare measures. A *p-*value ≤ 0.05 was considered statistically significant. Intra-class Correlation Coefficient (ICC), Passing-Bablok regression and Bland-Altman plots were used to evaluate the intra- and inter-observers concordance of HRCTV_MR_ and HRCTV_TRUS_ maximum width measurement.

ICC is a test of concordance or agreement for continuous data and ranges from 0 to 1 [28]. The concept of concordance is that the values obtained in different measurements are identical. With ICC we handle intra- as well inter- observer reproducibility of measurements corresponding to a fixed set of three judges rating each target [29]. In this work an ICC value inferior to 0.4 represent poor agreement, values in between 0.4 and 0.75 represent fair to good agreement and values above 0.75 represent excellent agreement [30].

Passing-Bablok regression method is based on non-parametric model. Result of Passing and Bablok regression consists of several parts and each has its role in interpreting data and concluding on methods agreement. The first result is scatter diagram with regression line that enables visual inspection of measured data and obvious agreement of fitted regression line and identity line. Regression equation (y = a + bx) reveals constant (regression line’s intercept (a)) and proportional (regression line’s slope (b)) difference with their confidence intervals of 95% (95% CI). Confidence intervals explain if their value differ from value zero for intercept and value one for slope only by chance. Thus, if 95% CI for intercept includes value zero it can be concluded that there is no significant difference between obtained intercept value and value zero and there is no constant difference between two methods. Respectively, if 95% CI for slope includes value one, it can be concluded that there is no significant difference between obtained slope value and value one and there is no proportional difference between two methods. [31].

Bland-Altman residual-like plots are a graphic representation of the data, with the difference between tested measures plotted against their mean values. They define limits of agreement by combining the mean (d) and standard deviations (s) of the differences as d + 2 s [32].

## Results

One hundred ten consecutive cervical cancer patients were analyzed. HRCTV_MR_ and HRCTV_TRUS_ average measures ± standard deviations (SD) are given in Table 2.

**Table 2:** Tumor maximum width average measures

	HRCVT_MR_			HRCTV_TRUS_		
	Obs. 1	Obs. 2	Obs. 1	Obs. 2	Obs. 1	Obs. 2
All stages	45.2 ± 12.7	43.6 ± 12.6	43.7 ± 12.2	43.9 ± 11.4	42.5 ± 11.9	43 ± 11.2
FIGO I	37.4 ± 7.7	37.4 ± 9.2	36.1 ± 8.8	36.3 ± 7.3	36.9 ± 8.7	36.5 ± 8.4
FIGO II	41.6 ± 8.4	39.7 ± 7.9	40.4 ± 7.9	40.6 ± 7.3	39.2 ± 8	39.6 ± 6.8
FIGO III	60.2 ± 11.3	58.2 ± 12.4	58.1 ± 11.7	57.9 ± 10.1	54.7 ± 12.9	57 ± 10.7
FIGO IV	63.7 ± 11.7	61.5 ± 11.2	60.1 ± 11.8	61.2 ± 9.4	58.3 ± 12.5	57.9 ± 11.2

Tumor maximum width average measures in mm (± standard deviations) measured in PreBT MR imaging (HRCTV_MR_) and in PreBT TRUS imaging acquired at the time of first brachytherapy application with no applicator in place (HRCTV_TRUS_), presented by FIGO (International Federation of Gynaecology and Obstetrics) stages. (*Abbreviations*: *Obs.* Observer)

### Intra-observer analysis

ICC analysis (Table 3) shows an excellent agreement between HRCTV_TRUS_ and HRCTV_MR_ maximum width for all three observers. ICC values were slightly lower for larger tumors. Passing-Bablok regression and Bland-Altman plots confirm this data (Additional file 1).

**Table 3:** Intra-observer agreement analysis of tumor maximum width measurements

	HRCVT_MR_ vs. HRCTV_TRUS_		
		ICC	CI 95%
All stages	Obs. 1	0.96	[0.94–0.97]
	Obs. 2	0.93	[0.90–0.95]
	Obs. 3	0.96	[0.95–0.98]
FIGO I	Obs. 1	0.95	[0.87–0.98]
	Obs. 2	0.98	[0.94–0.99]
	Obs. 3	0.97	[0.92–0.99]
FIGO II	Obs. 1	0.94	[0.9–0.96]
	Obs. 2	0.97	[0.95–0.98]
	Obs. 3	0.9	[0.85–0.94]
FIGO III	Obs. 1	0.91	[0.75–0.97]
	Obs. 2	0.7	[0.3–0.89]
	Obs. 3	0.98	[0.94–0.99]
FIGO IV	Obs. 1	0.87	[0.57–0.96]
	Obs. 2	0.82	[0.44–0.95]
	Obs. 3	0.93	[0.76–0.98]

Note that: ICC > 0,75: excellent agreement; 0,4 – 0,75: fair to good agreement; < 0,4: poor agreement

*Abbreviations*: *HRCTV*_*MR*_ High risk clinical target volume maximum width measured at preBT MR, *HRCTV*_*TRUS*_ Tumor maximum width measured at preBT TRUS, *ICC* Intra-class Correlation Coefficient, *CI* Confidence interval, *FIGO* International Federation of Gynaecology and Obstetrics, *Obs* Observer

Further, a quantitative analysis was undertaken in order to determine the magnitude of the uncertainties between HRCTV_MR_ (considered as gold standard) and HRCTV_TRUS_ width measurements. Overall average difference between HRCTV_MR_ and HRCTV_TRUS_ was 1.3 ± 3.2 mm (*p < 0.001*); 1.1 ± 4.6 mm (*p = 0.01*) and 0.7 ± 3 mm (*p = 0.01*) for the three observers, respectively. For FIGO stage I the average difference was 1.1 ± 2.2 mm (*p = 0.024*), 0.5 ± 2 mm (*p = NS*) and − 0.4 ± 2.1 mm (*p = NS*). For FIGO stage II it was 1 ± 2.7 mm (*p = 0.004*), 0.5 ± 1.9 mm (*p = 0.046*) and 0.7 ± 3.2 mm (*p = NS*). For FIGO stage III the average difference between HRCTV_MR_ and HRCTV_TRUS_ was 2.3 ± 4.2 mm (*p = NS*); 3.5 ± 9.6 mm (*p = NS*) and 1.1 ± 2 mm (*p = NS*). Finally for FIGO stage IV the difference was 2.5 ± 5.2 mm (*p = NS*); 3.1 ± 7.3 mm (*p = NS*) and 2.2 ± 4 mm (*p = NS*) for the three observers respectively.

We defined differences between HRCTV_MR_ and HRCTV_TRUS_ of less than 3 mm as minor, between 3 and 5 mm as potentially relevant and of more than 5 mm as major.

In the whole cohort of 110 patients (pts), the difference between HRCTV_MR_ and HRCTV_TRUS_ measures were less than 3 mm in 87 pts. (79%) for observer 1, in 90 pts. (82%) for observer 2 and in 88 pts. (80%) for observer 3. It was between 3 and 5 mm: in 10 pts. (9%) for observer 1 and 2, and in 11 pts. (10%) for observer 3. It was more than 5 mm in 13 pts. 12% for observer 1, in 10 pts. (9%) for observer 2 and in 11 pts. (10%) for observer 3. TRUS was more likely to underestimate tumor width in large FIGO IIIB and IVA tumors (Additional file 1: Figures S2.1 and S2.2).

### Inter-observer analysis

Inter-observer agreement of HRCTV_TRUS_ and HRCTV_MR_ maximum width measures was calculated with ICC. As expected, overall agreement of HRCTV_MR_ measures was 0.92 (CI95% 0.89–0.94) with no differences among FIGO stages. Overall HRCTV_TRUS_ measure consistency was 0.86 (CI95% 0.81–0.9). ICC value for HRCTV_TRUS_ was comparable to HRCTV_MR_ in FIGO stages I and II tumors and progressively decreased in larger tumors (FIGO stage III and IV), although remaining fairly good (Table 4). In a one-to-one comparison, Passing-Bablok regression and Bland-Altman plots confirmed no substantial differences among individual observers (Additional file 1: Figure S3).

**Table 4:** Inter-observer agreement analysis of tumor maximum width measurements

	HRCVT_MR_		HRCTV_TRUS_	
	ICC	CI 95%	ICC	CI 95%
All stages	0.92	[0.89–0.94]	0.86	[0.81–0.9]
FIGO I	0.87	[0.75–0.94]	0.88	[0.76–0.95]
FIGO II	0.81	[0.74–0.87]	0.79	[0.7–0.86]
FIGO III	0.88	[0.75–0.96]	0.69	[0.42–0.87]
FIGO IV	0.93	[0.83–0.98]	0.62	[0.26–0.87]

Note that: ICC > 0,75: excellent agreement; 0,4 – 0,75: fair to good agreement; < 0,4: poor agreement

*Abbreviations*: *HRCTV*_*MR*_ High risk clinical target volume maximum width measured at preBT MR, *HRCTV*_*TRUS*_ Tumor maximum width measured at preBT TRUS, *ICC* Intra-class Correlation Coefficient, *CI* Confidence interval, *FIGO* International Federation of Gynaecology and Obstetrics

## Discussion

In the last few years US-based BT dose adaptation has been increasingly investigated for cervical cancer IGABT [33]. US has been proven as an excellent diagnostic imaging modality in gynecological oncology [34] and it has been extensively used during BT application to guide tandem and needles insertion [35].

One of the largest prospective studies comparing the diagnostic accuracy of TRUS and MR in the local staging of cervical cancer was published by Fischerova et al. [36] in 2008 and included 95 patients with early-stage disease. The study showed a significantly higher accuracy of TRUS when compared to MR in tumor identification (considering also residual tumor after previous biopsy (93.7 vs. 83.2%, *p* ≤ 0.006), or small tumors ≤1 cm^3^ (90.5 vs. 81%, *p* ≤ 0.049)). Similar results were demonstrated by a European multicenter prospective study, which included 182 patients with histologically confirmed early-stage cancer. The diagnostic agreement between ultrasound and pathology was significantly better at detecting residual tumor and parametrial invasion than MR (*p* < 0.001). A surprising finding was the maintenance of diagnostic accuracy of ultrasound in the detection of residual tumor after cone biopsy, where it is difficult to distinguish post-inflammatory and reparative changes after the procedure from the presence of residual tumor [37].

Pinkakova et al. in a prospective study on a cohort of 42 FIGO IB1-IIB cervical cancer patients (with limited parametrial involvement) demonstrated the non- inferiority of TRUS with respect to MR in assessing tumor regression during neo-adjuvant chemotherapy [38].

The potential of TRUS imaging in tracking the tumor changes and regression during EBRT is a fact of critical relevance if TRUS is used to guide the BT insertion (normally scheduled after 3–4 weeks of radio-chemotherapy) and, potentially, dose adaptation.

The clinical use of US-based BT dose adaptation in cervical cancer BT has been pioneered at Peter MacCallum Cancer Center [39] and promising results have been reported [40]. The method suggested is based on transabdominal US measurements of the uterus taken along the tandem axis in the sagittal plan and has shown a robust correlation with MR measurement. This approach is useful to conform the dose distribution according uterus silhouette in the anteroposterior diameter thus reducing bladder and rectal dose. Nevertheless it seems unfit to target volume delineation at the parametria level, because the limitations of transabdominal US in detecting parametrial invasion and the unfeasibility of true volumetric image acquisition. For all these reasons, Kirisits et al. stated, in an interesting editorial, “this method may be useful, mainly in limited size and well-responding tumors, which are confined to the cervix at the time of BT. However, this clinical scenario does not represent most patients in advanced stage as seen in the countries with high patient numbers and limited resources” [33].

Conversely, TRUS (which is already extensively used for prostate BT imaging and dose optimization) allows a true volumetric image acquisition and a detailed imaging of cervical tumor and eventual extension beyond uterine cervix within the parametrial space [41]. Parametrial infiltration is a well known prognostic parameter for cervical cancer [42] and probably the most relevant factor to take into account at the time of IGABT insertion preplanning [43], to select between intracavitary or intracavitary/interstitial technique [44]. For this reason, the correct assessment of tumor width is a critical point in cervical cancer BT. Validation of TRUS as potentially useful tool for cervical BT imaging and dose adaptation starts from the assessment of the robustness of TRUS measuring tumor width at the time of BT.

Researchers at the Medical University of Vienna in two different studies [25, 45] demonstrated an excellent agreement between MR and TRUS in assessing tumor maximum width after EBRT in respectively 16 and 19 patients diagnosed with cervical cancers (FIGO I-IV). In both studies the mean difference between MR and TRUS measurement were in the same range (− 0.3 ± 3.2 mm and − 1.1 ± 3.2 mm respectively). These data strikingly compare with our results (1.3 ± 3.2 mm; 1.1 ± 4.6 mm and 0.7 ± 3 mm for the 3 observers). We were also able to demonstrate that the magnitude of uncertainty in the vast majority of cases (≈80%) is very small (< 3 mm), but increases in large tumors. Additionally, we demonstrated that in large IIIB/IVA tumors TRUS may underestimate tumor width compared to MR. Several reasons might be claimed to explain such data.

First of all, the higher degree of uncertainty associated to tumor maximum width measurement in large tumors that spread to the pelvic wall. In fact, even if the maximum tumor width in this study is taken perpendicularly to the cervical canal and uterine axis (in order to improve reproducibility of the measurements between MR and TRUS), the irregular shape of such tumors may impair the identification of a reproducible axis of measurement (Additional file 1: Figure S4). This type of error may be at the basis of the progressively lower TRUS concordance among observers in advanced FIGO stage tumors. On the other hand, such type of uncertainty should be stochastic, while our findings show that TRUS underestimates tumor width (> 5 mm) in 20% of stage IV and in 7–14% of stage III tumors (Additional file 1: Figure S2.2).

A second possible explanation could be an intrinsic limited resolution of TRUS imaging in depicting the limit of tumor infiltration into pelvic wall, because poor image quality (due to patient preparation, or because TRUS shortcoming in evaluating the relationship of extensive tumors to the pelvic sidewall when the edge of infiltration is close to the range of the probe). During this investigation, a strict bowel preparation protocol was followed in order to prevent the interference on TRUS image quality. Nevertheless, TRUS performance in large tumors was inferior to MR, as shown by the inter-observer analysis, where the measurement agreement as expressed by ICC values was extremely high for HRCTV_MR_ width, independently from FIGO stage. Conversely, ICC for HRCTV_TRUS_ width resulted comparable to MR just for FIGO I/II tumors and progressively declined for larger tumors, although remaining fairly good (Table 4).

**Fig. 2:**
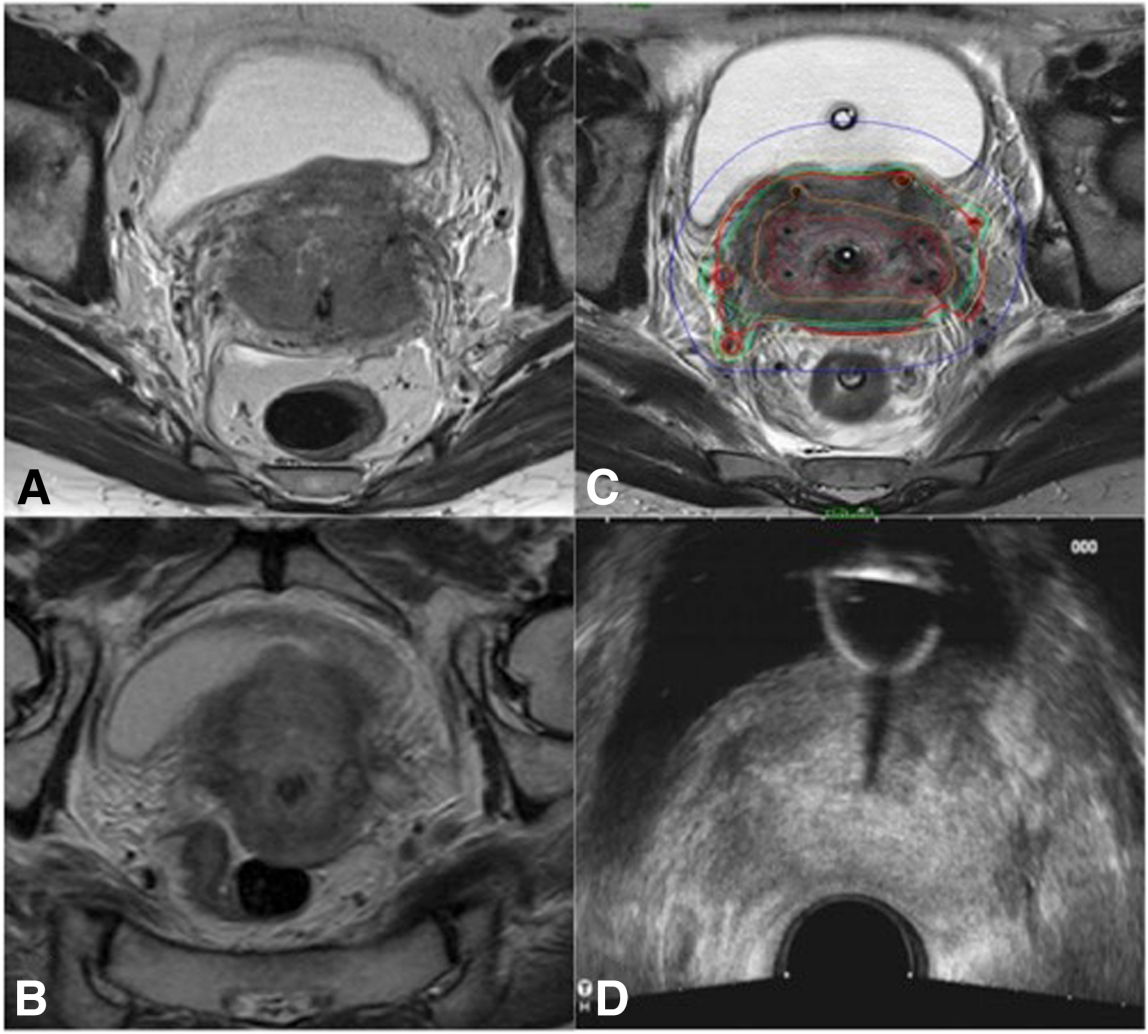
A case of cervical cancer FIGO stage IVA because bladder infiltration with poor response at EBRT with good agreement between preBT MR and preBT TRUS in tumor measures. Tumor at the time of diagnostic MR (**a**), at the time of preBT MR (**b**), at time of first brachytherapy (**c**) and at time the preBT TRUS (**d**)

**Fig. 3:**
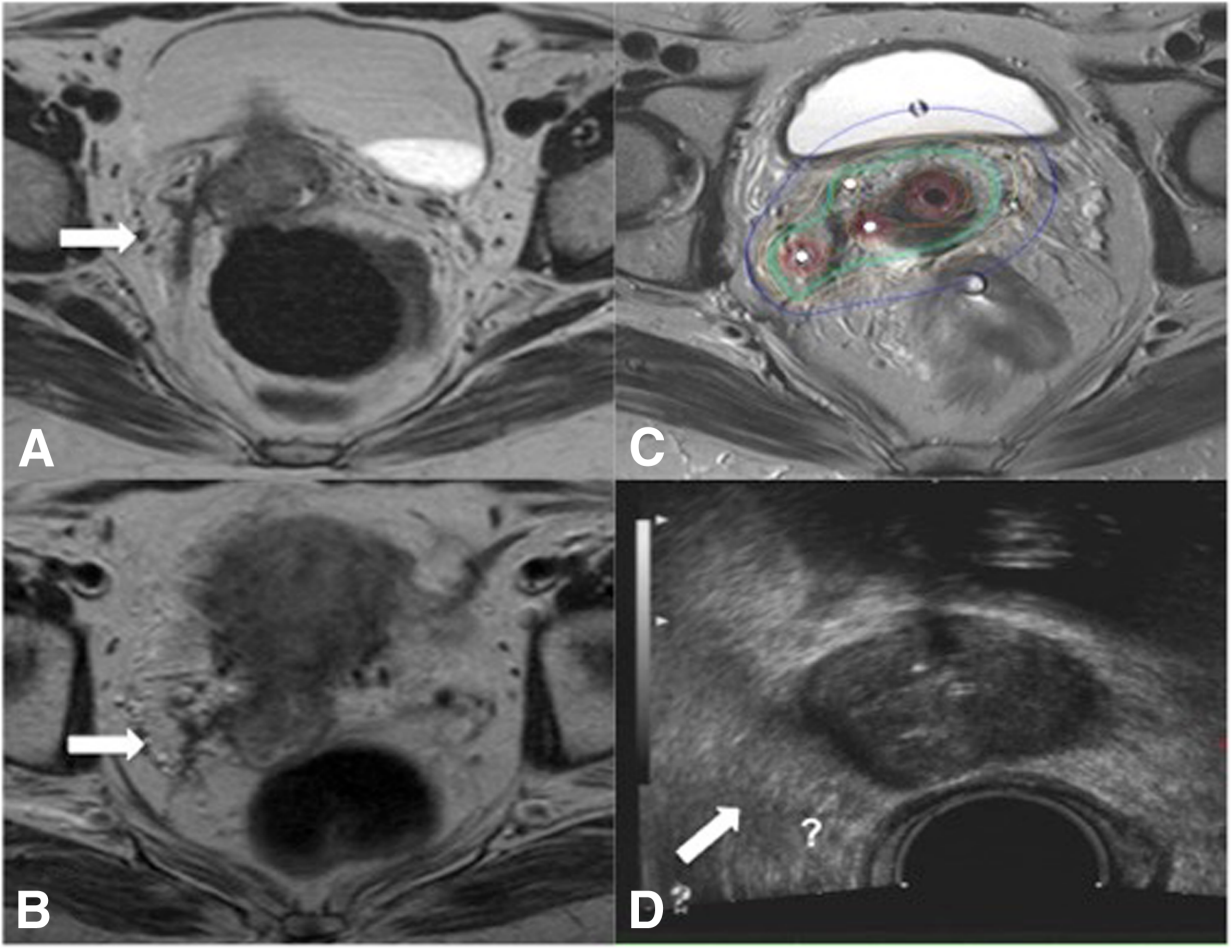
A case of cervical cancer FIGO stage IIIB with limited response to EBRT with bad agreement between pre BT MR and preBT TRUS in tumor measures. Tumor at the time of diagnostic MR (**a**), at time of preBT MR (**b**) and at time of first BT (**c**). In the preBT TRUS (**d**) the right parametrial invasion (white arrows) is not as clearly visible as in MR (**a**-**b**-**c**)

A further reason might depend on the shape and type of tumor growth pattern. Very advanced tumor stages (FIGO IVA) show more likely an infiltrative growth with irregular shapes, and thin digitations that deeply infiltrate the parametrial space in comparison to bulky expansive cervical tumors, which are generally more clearly visible in TRUS imaging (Figs. 2 and 3).

It deserves to be mention that a limitation of our study design and a potential bias is that TRUS images were acquired by one investigator only and then analyzed independently by the three investigators. To obtain three sets of TRUS images acquired independently by each observer would be probably more correct. Nevertheless, such study design would be impossible at our Institution because the organization of the workflow. To minimize uncertainties the TRUS acquisition, a protocol (previously described) was thoroughly followed.

Our investigation does not include the assessment of tumor thickness and height. Schmid et al. [25, 45] analyzed tumor thickness as measured by TRUS or MR and found a statistically significant underestimation with TRUS. Such underestimation, in their opinion, is mainly due to the compression of the cervix by the TRUS probe at the moment of image acquisition. We agree with this point. We believe that the insertion of rectal probe (and the angle needed to have it parallel to the uterus) may intrinsically bias any comparison with images taken with relaxed rectum/pelvic floor. On the other hand, the exact burden of tumor cranial infiltration into myometrium (especially after EBRT) is challenging on TRUS imaging (but also on MR). For this reason, in the clinic is generally recommended to load the tandem up to the *fundus uteri*, making the exact tumor height measurement in MR or TRUS not a critical point.

## Conclusions

Taken together our results suggest that TRUS is comparable to MR in assessing preBT tumor maximum width in cervical cancer FIGO stage I/II. In more advance stages TRUS seems to be slightly inferior to MR although maintaining a good agreement to gold standard imaging.

Given the limited cost of TRUS compared to MR and the potential of improved patient accessibility, especially in low income countries, TRUS based IGABT is a major field of research in radiation oncology. Further studies are however still needed to define the technical modality of integration of TRUS in cervical cancer IGABT and TRUS-based dose adaptation.

## Additional file

Additional file 1 (PPT 3891 kb)

## Data Availability

The datasets used and analysed during the current study (database with measures) are available from the corresponding author on reasonable request.

## Abbreviations

BT: Brachytherapy
CI: Confidence interval
CT: Computer tomography
EBRT: External beam radiotherapy
HRCTV: High risk clinical target volume
ICC: Intra-class correlation coefficient
IGABT: Image guided adapted brachytherapy
MR: Magnetic resonance
OAR: Organs at risk
RT: Radiotherapy
SD: Standard deviation
TRUS: Trans rectal Ultrasound
