# Supplementary material for: Prospective intra/inter-observer evaluation of pre-brachytherapy cervical cancer tumor width measured in TRUS and MR imaging
Source: Radiat Oncol. 2019 Oct 4;14:173. doi: 10.1186/s13014-019-1352-7 (PMC6778388; doi:10.1186/s13014-019-1352-7)
Supplement: Supplementary file 1 — (PPT 3891 kb) [file 13014_2019_1352_MOESM1_ESM.ppt]

## Slide 1
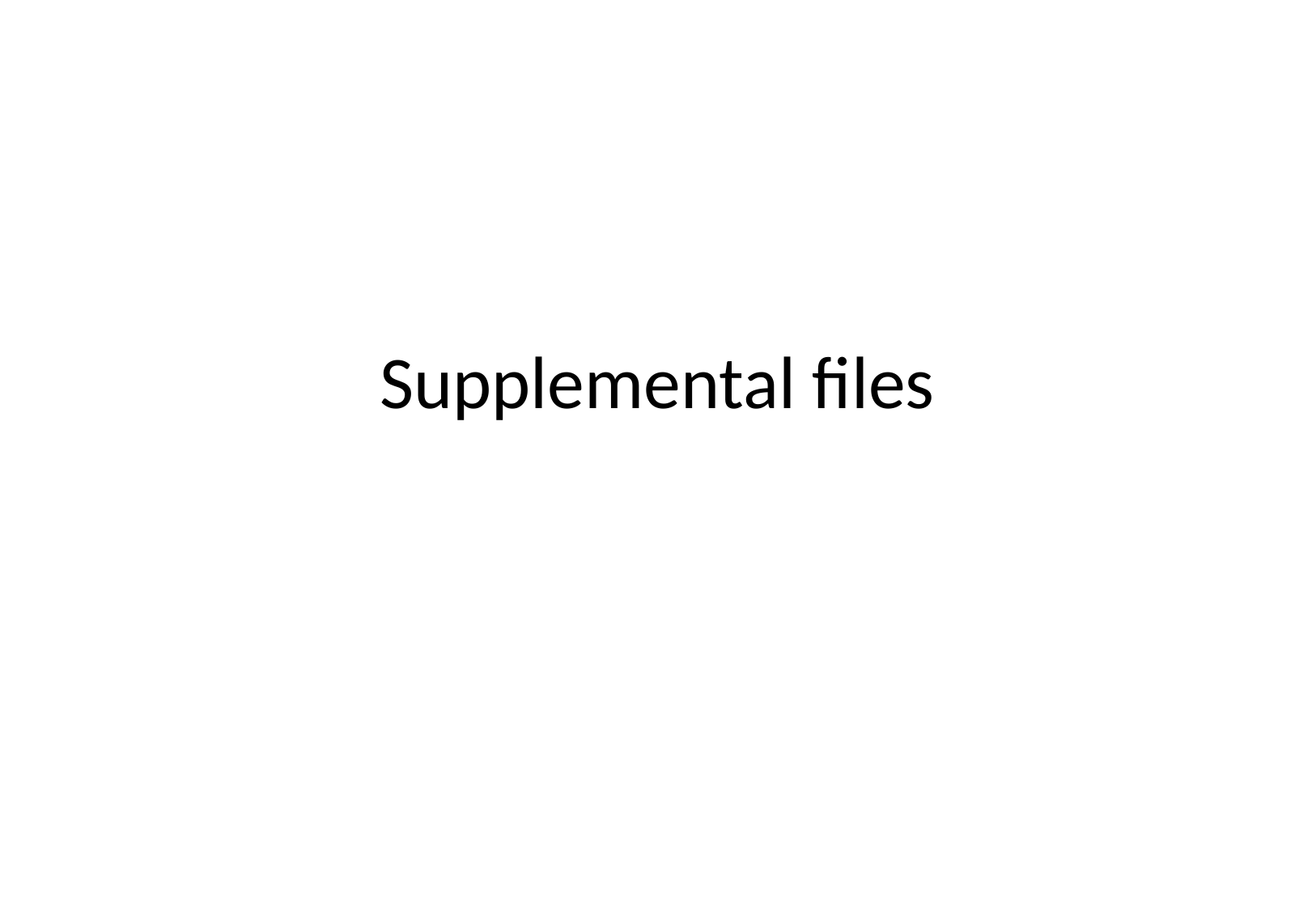

# Supplemental files

## Slide 2
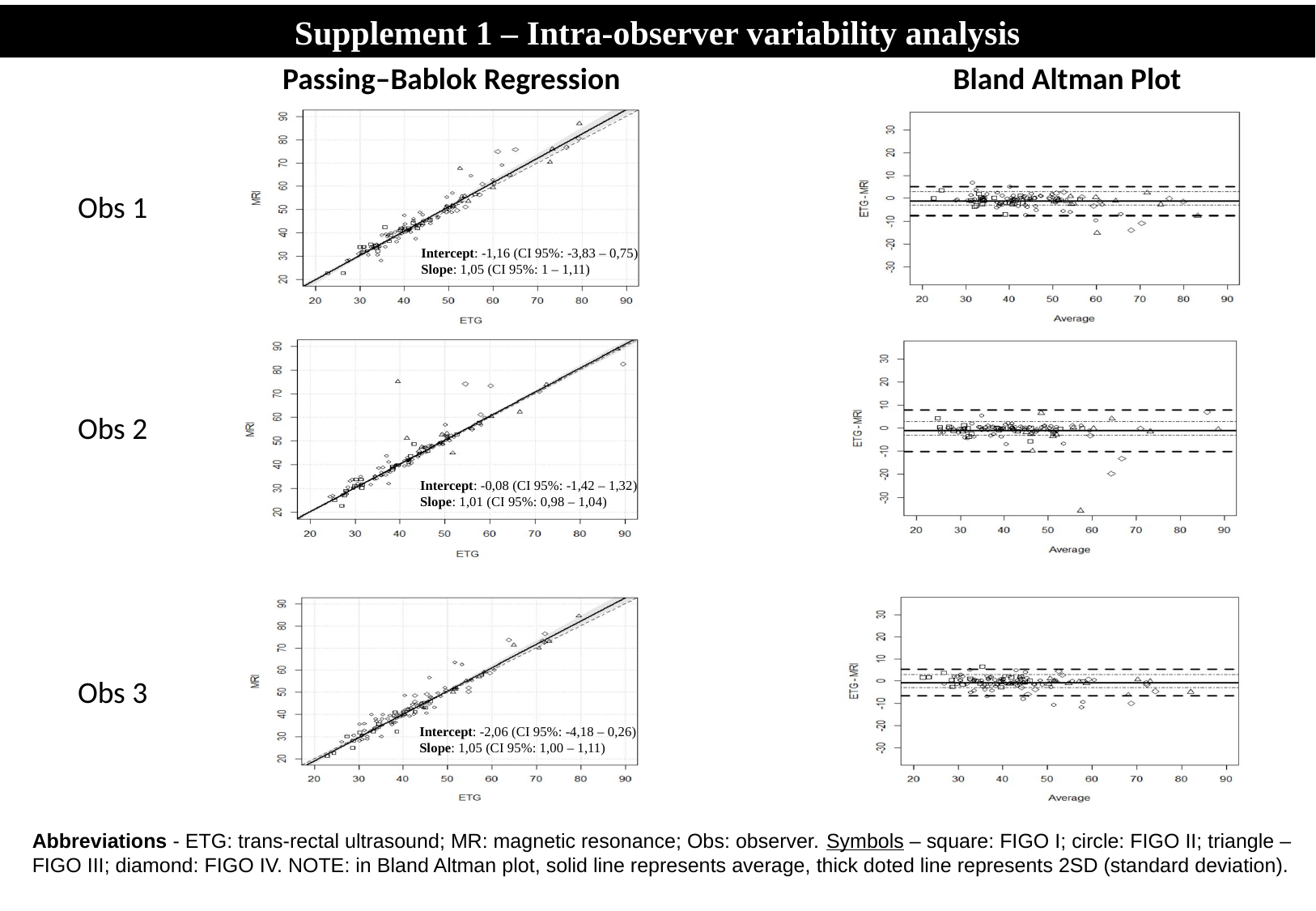

Supplement 1 – Intra-observer variability analysis
Passing–Bablok Regression
Bland Altman Plot
Obs 1
Intercept: -1,16 (CI 95%: -3,83 – 0,75)
Slope: 1,05 (CI 95%: 1 – 1,11)
Obs 2
Intercept: -0,08 (CI 95%: -1,42 – 1,32)
Slope: 1,01 (CI 95%: 0,98 – 1,04)
Obs 3
Intercept: -2,06 (CI 95%: -4,18 – 0,26)
Slope: 1,05 (CI 95%: 1,00 – 1,11)
# Abbreviations - ETG: trans-rectal ultrasound; MR: magnetic resonance; Obs: observer. Symbols – square: FIGO I; circle: FIGO II; triangle – FIGO III; diamond: FIGO IV. NOTE: in Bland Altman plot, solid line represents average, thick doted line represents 2SD (standard deviation).

## Slide 3
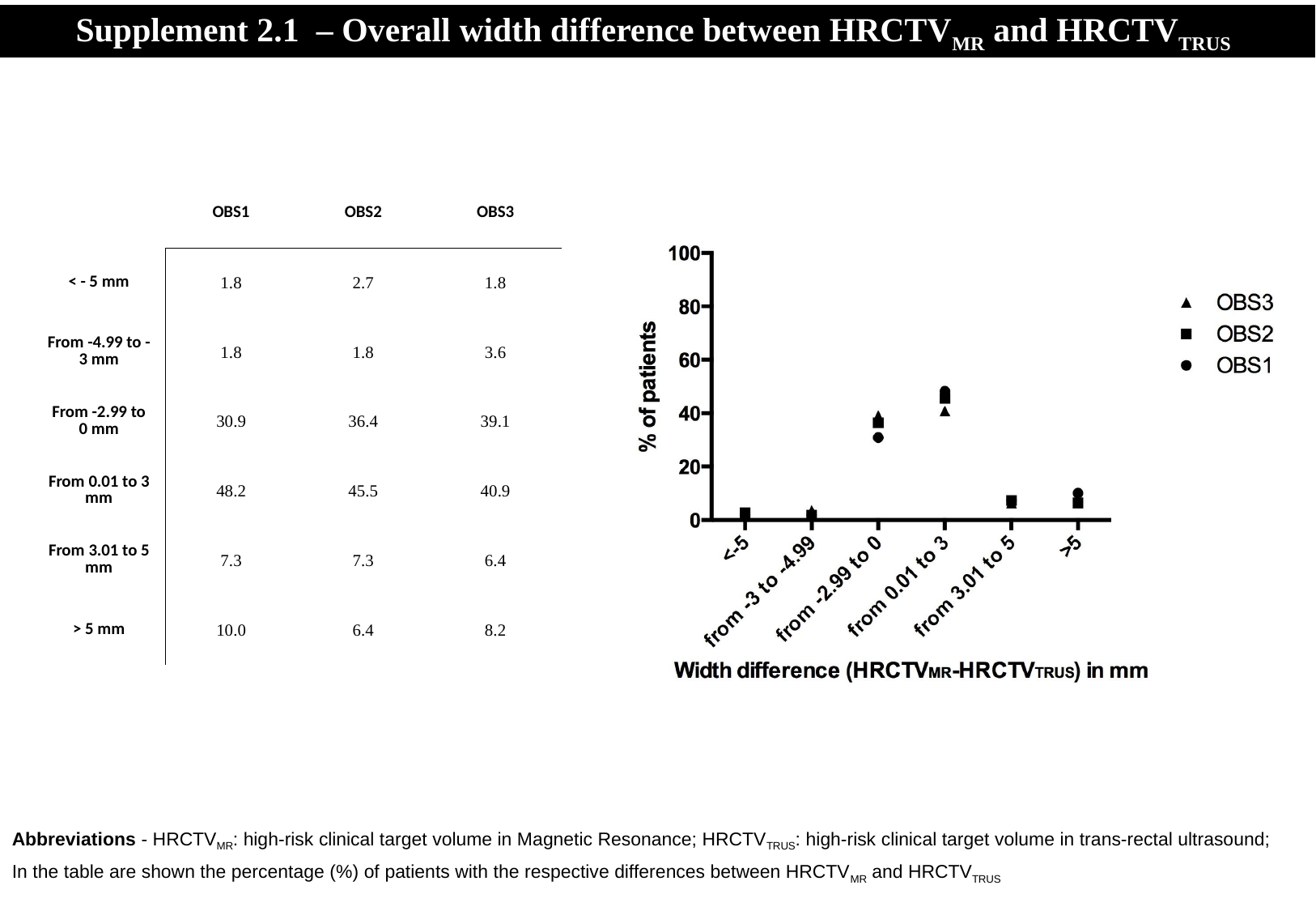

Supplement 2.1 – Overall width difference between HRCTVMR and HRCTVTRUS
| | OBS1 | OBS2 | OBS3 |
| --- | --- | --- | --- |
| < - 5 mm | 1.8 | 2.7 | 1.8 |
| From -4.99 to -3 mm | 1.8 | 1.8 | 3.6 |
| From -2.99 to 0 mm | 30.9 | 36.4 | 39.1 |
| From 0.01 to 3 mm | 48.2 | 45.5 | 40.9 |
| From 3.01 to 5 mm | 7.3 | 7.3 | 6.4 |
| > 5 mm | 10.0 | 6.4 | 8.2 |
Abbreviations - HRCTVMR: high-risk clinical target volume in Magnetic Resonance; HRCTVTRUS: high-risk clinical target volume in trans-rectal ultrasound; In the table are shown the percentage (%) of patients with the respective differences between HRCTVMR and HRCTVTRUS

## Slide 4
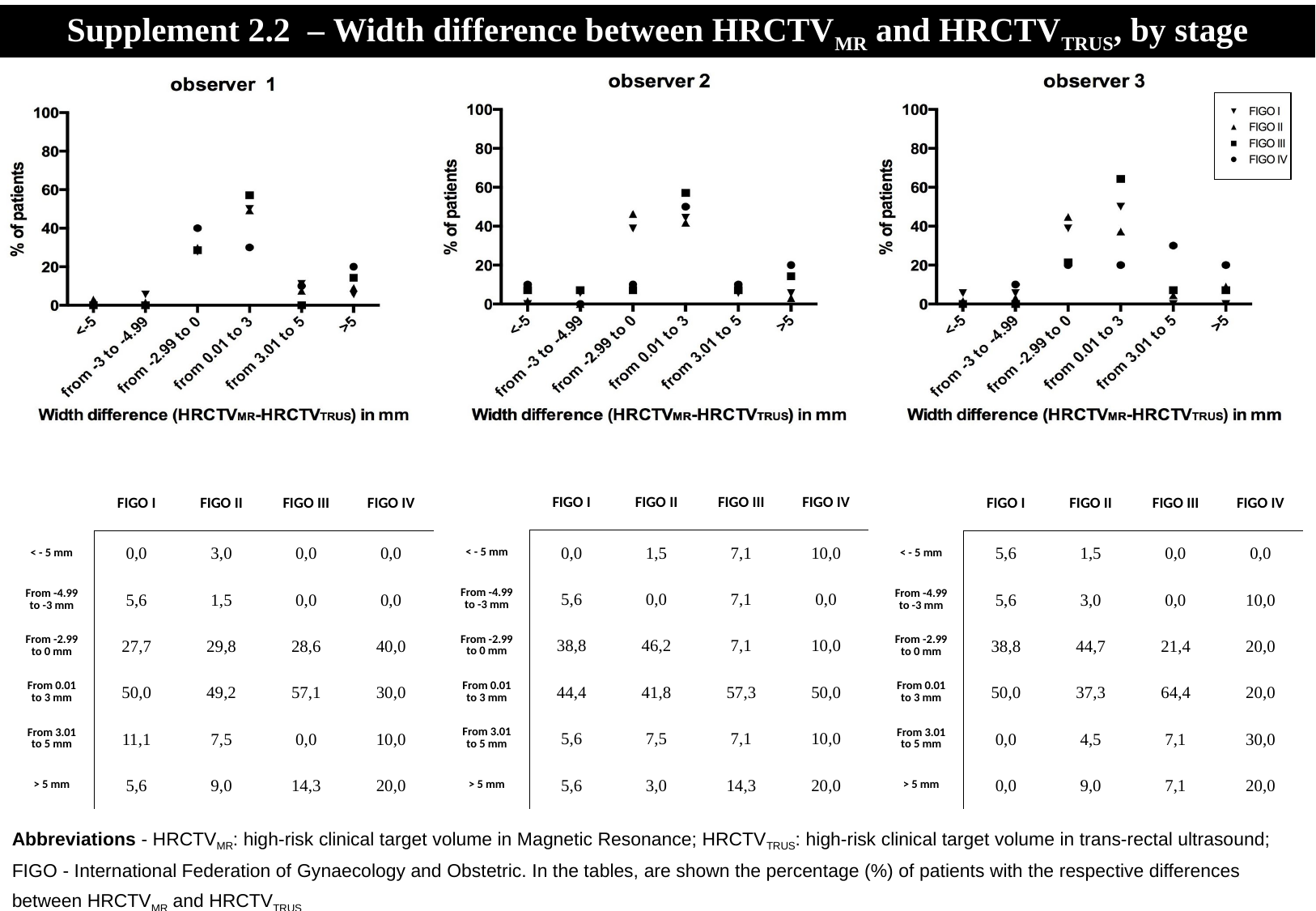

Supplement 2.2 – Width difference between HRCTVMR and HRCTVTRUS, by stage
| | FIGO I | FIGO II | FIGO III | FIGO IV |
| --- | --- | --- | --- | --- |
| < - 5 mm | 0,0 | 1,5 | 7,1 | 10,0 |
| From -4.99 to -3 mm | 5,6 | 0,0 | 7,1 | 0,0 |
| From -2.99 to 0 mm | 38,8 | 46,2 | 7,1 | 10,0 |
| From 0.01 to 3 mm | 44,4 | 41,8 | 57,3 | 50,0 |
| From 3.01 to 5 mm | 5,6 | 7,5 | 7,1 | 10,0 |
| > 5 mm | 5,6 | 3,0 | 14,3 | 20,0 |
| | FIGO I | FIGO II | FIGO III | FIGO IV |
| --- | --- | --- | --- | --- |
| < - 5 mm | 0,0 | 3,0 | 0,0 | 0,0 |
| From -4.99 to -3 mm | 5,6 | 1,5 | 0,0 | 0,0 |
| From -2.99 to 0 mm | 27,7 | 29,8 | 28,6 | 40,0 |
| From 0.01 to 3 mm | 50,0 | 49,2 | 57,1 | 30,0 |
| From 3.01 to 5 mm | 11,1 | 7,5 | 0,0 | 10,0 |
| > 5 mm | 5,6 | 9,0 | 14,3 | 20,0 |
| | FIGO I | FIGO II | FIGO III | FIGO IV |
| --- | --- | --- | --- | --- |
| < - 5 mm | 5,6 | 1,5 | 0,0 | 0,0 |
| From -4.99 to -3 mm | 5,6 | 3,0 | 0,0 | 10,0 |
| From -2.99 to 0 mm | 38,8 | 44,7 | 21,4 | 20,0 |
| From 0.01 to 3 mm | 50,0 | 37,3 | 64,4 | 20,0 |
| From 3.01 to 5 mm | 0,0 | 4,5 | 7,1 | 30,0 |
| > 5 mm | 0,0 | 9,0 | 7,1 | 20,0 |
Abbreviations - HRCTVMR: high-risk clinical target volume in Magnetic Resonance; HRCTVTRUS: high-risk clinical target volume in trans-rectal ultrasound; FIGO - International Federation of Gynaecology and Obstetric. In the tables, are shown the percentage (%) of patients with the respective differences between HRCTVMR and HRCTVTRUS

## Slide 5
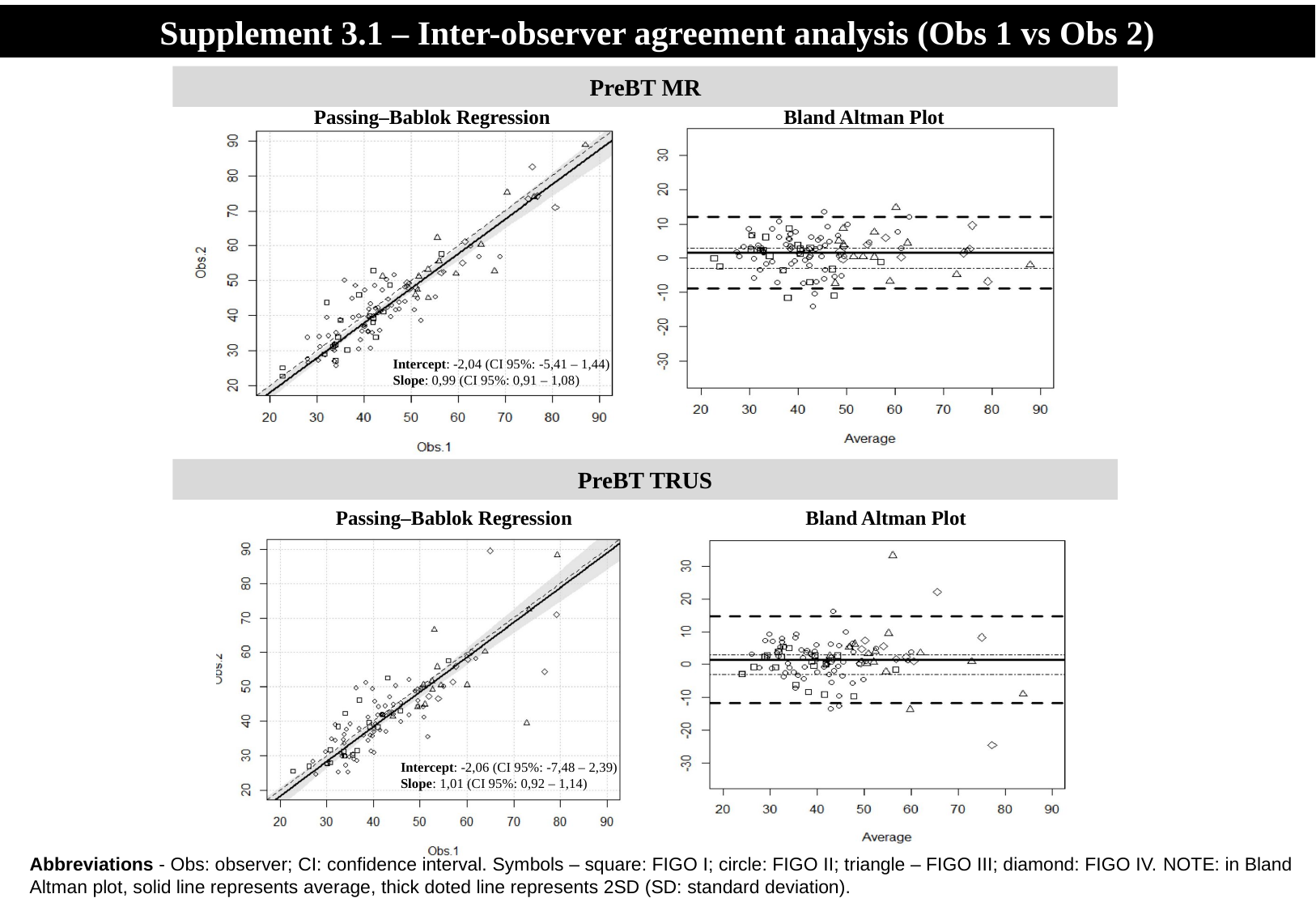

Supplement 3.1 – Inter-observer agreement analysis (Obs 1 vs Obs 2)
PreBT MR
Bland Altman Plot
Passing–Bablok Regression
Intercept: -2,04 (CI 95%: -5,41 – 1,44)
Slope: 0,99 (CI 95%: 0,91 – 1,08)
PreBT TRUS
Bland Altman Plot
Passing–Bablok Regression
Intercept: -2,06 (CI 95%: -7,48 – 2,39)
Slope: 1,01 (CI 95%: 0,92 – 1,14)
Abbreviations - Obs: observer; CI: confidence interval. Symbols – square: FIGO I; circle: FIGO II; triangle – FIGO III; diamond: FIGO IV. NOTE: in Bland Altman plot, solid line represents average, thick doted line represents 2SD (SD: standard deviation).

## Slide 6
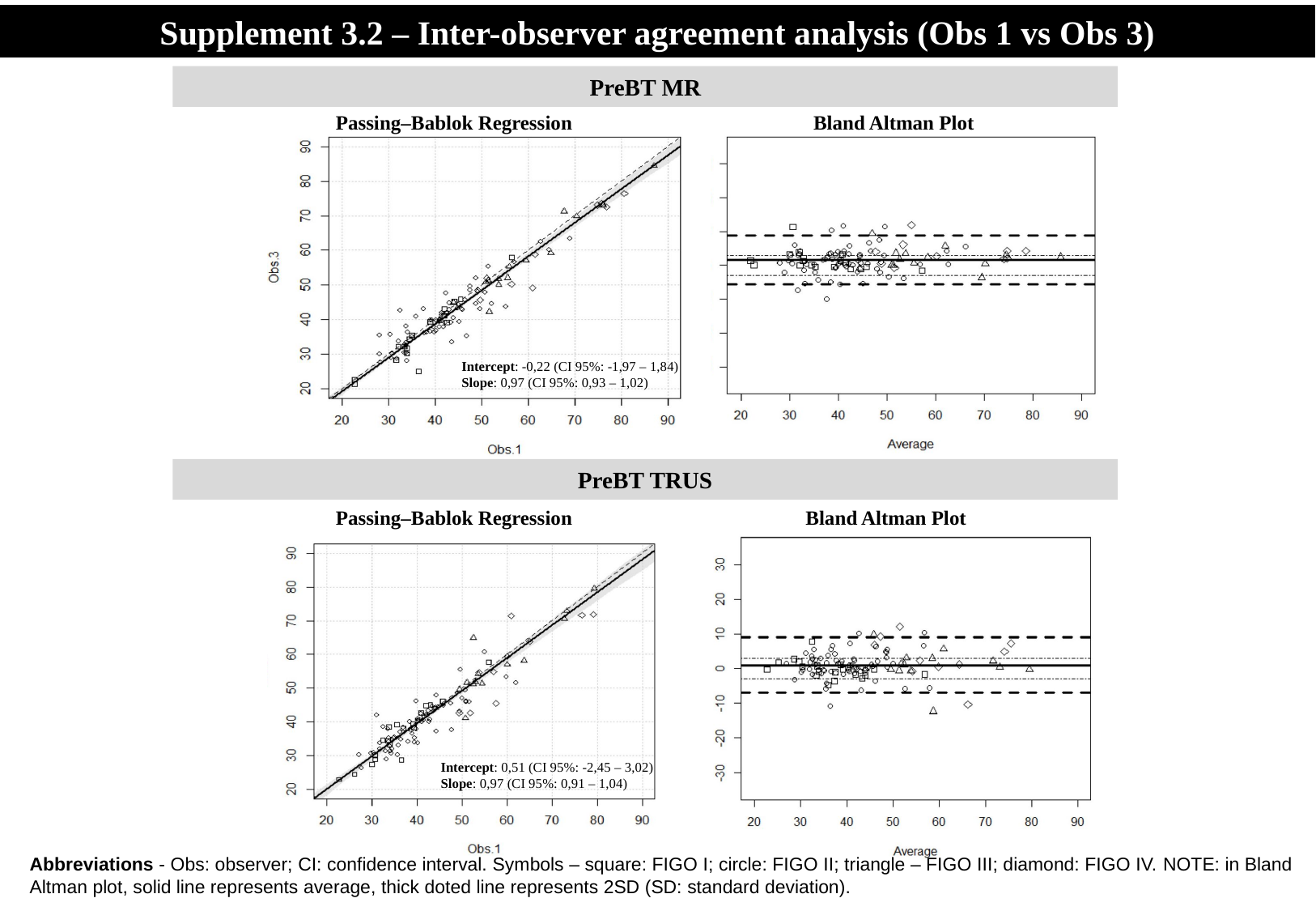

Supplement 3.2 – Inter-observer agreement analysis (Obs 1 vs Obs 3)
PreBT MR
Passing–Bablok Regression
Bland Altman Plot
Intercept: -0,22 (CI 95%: -1,97 – 1,84)
Slope: 0,97 (CI 95%: 0,93 – 1,02)
PreBT TRUS
Bland Altman Plot
Passing–Bablok Regression
Intercept: 0,51 (CI 95%: -2,45 – 3,02)
Slope: 0,97 (CI 95%: 0,91 – 1,04)
Abbreviations - Obs: observer; CI: confidence interval. Symbols – square: FIGO I; circle: FIGO II; triangle – FIGO III; diamond: FIGO IV. NOTE: in Bland Altman plot, solid line represents average, thick doted line represents 2SD (SD: standard deviation).

## Slide 7
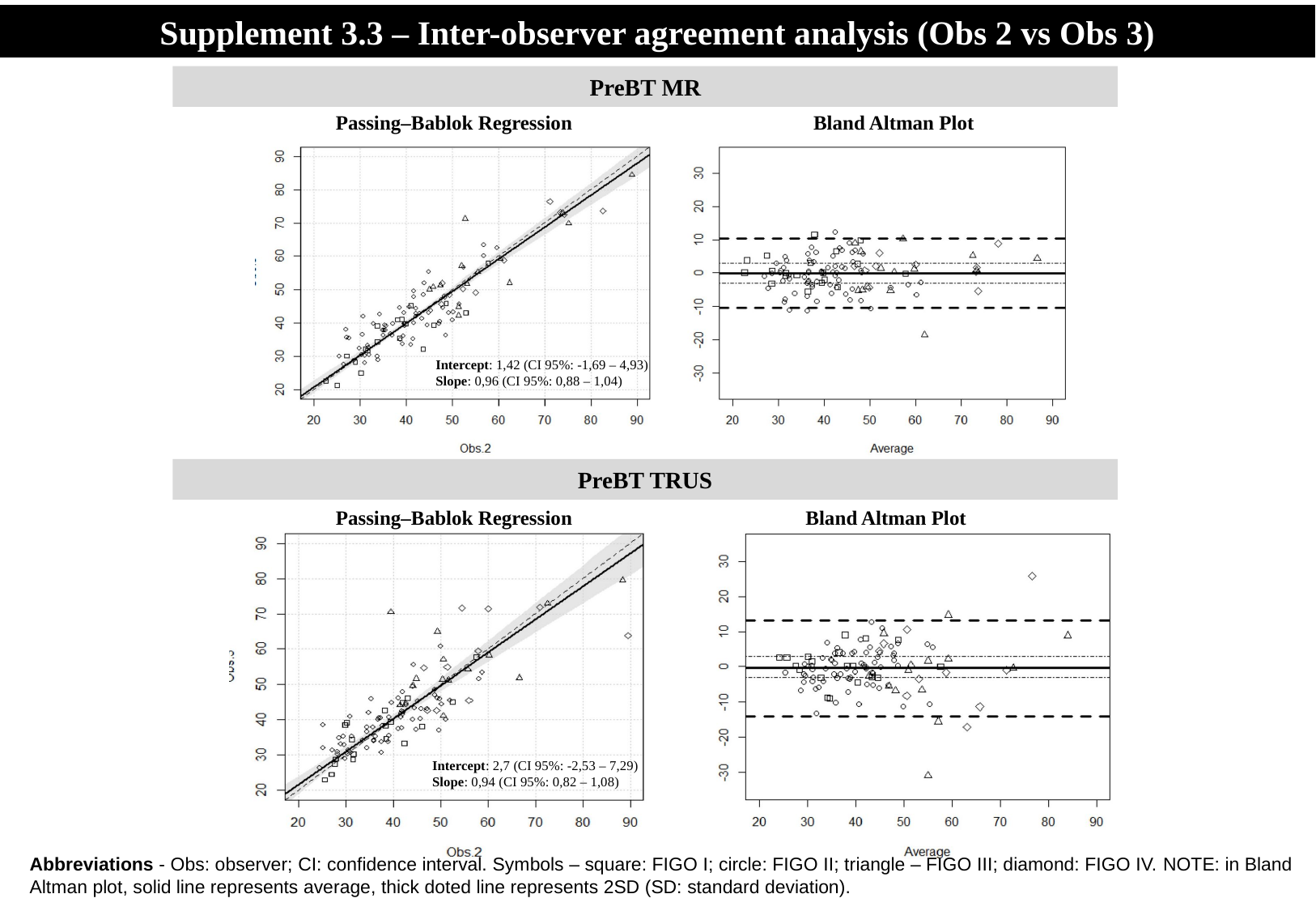

Supplement 3.3 – Inter-observer agreement analysis (Obs 2 vs Obs 3)
PreBT MR
Passing–Bablok Regression
Bland Altman Plot
Intercept: 1,42 (CI 95%: -1,69 – 4,93)
Slope: 0,96 (CI 95%: 0,88 – 1,04)
PreBT TRUS
Bland Altman Plot
Passing–Bablok Regression
Intercept: 2,7 (CI 95%: -2,53 – 7,29)
Slope: 0,94 (CI 95%: 0,82 – 1,08)
Abbreviations - Obs: observer; CI: confidence interval. Symbols – square: FIGO I; circle: FIGO II; triangle – FIGO III; diamond: FIGO IV. NOTE: in Bland Altman plot, solid line represents average, thick doted line represents 2SD (SD: standard deviation).

## Slide 8
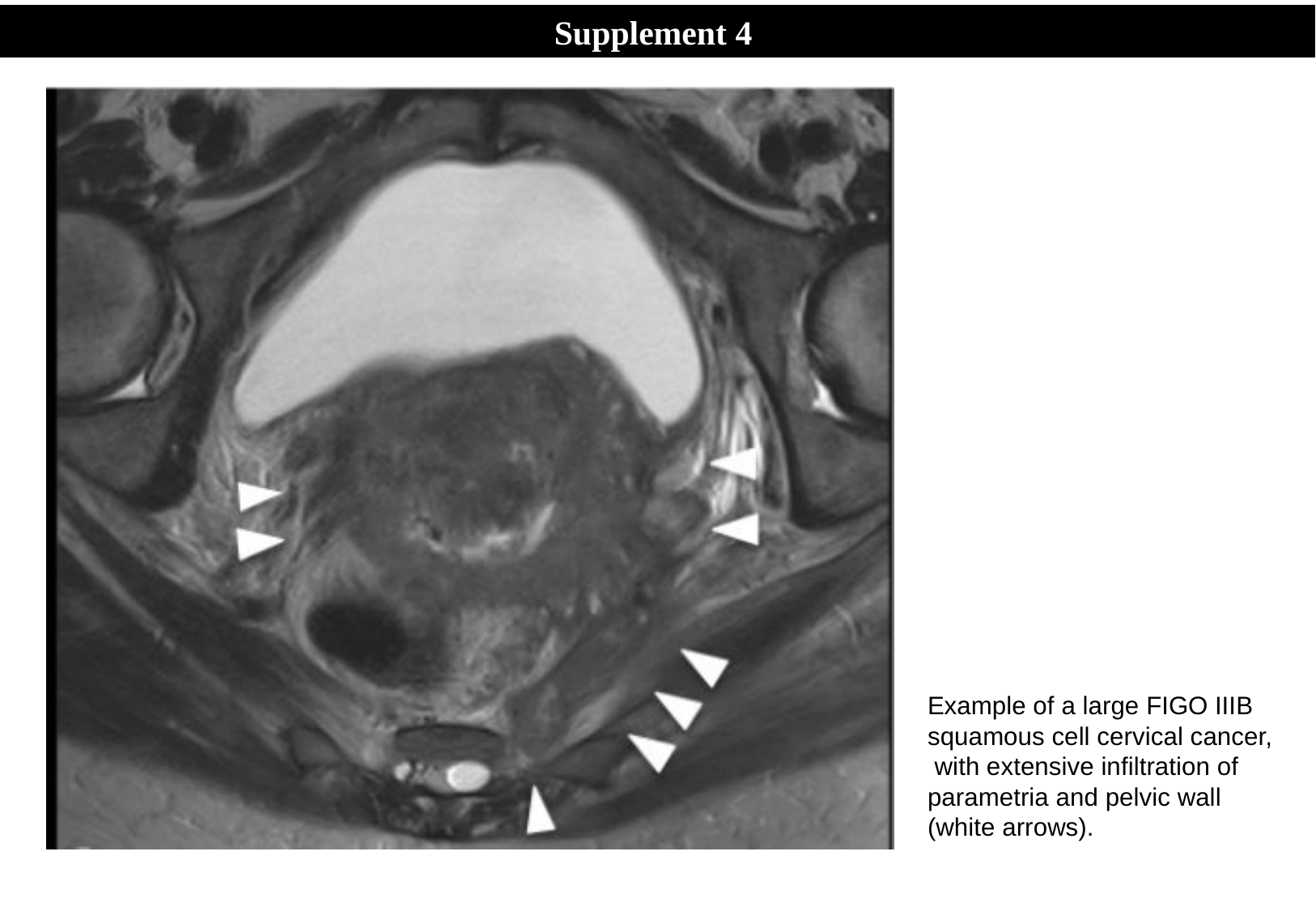

Supplement 4
Example of a large FIGO IIIB squamous cell cervical cancer, with extensive infiltration of parametria and pelvic wall (white arrows).
